# Postglacial change of the floristic diversity gradient in Europe

**DOI:** 10.1038/s41467-019-13233-y

**Published:** 2019-11-28

**Authors:** Thomas Giesecke, Steffen Wolters, Jacqueline F. N. van Leeuwen, Pim W. O. van der Knaap, Michelle Leydet, Simon Brewer

**Affiliations:** 1 Palaeoecology, Department of Physical Geography, Faculty of Geosciences, Utrecht University, P.O. Box 80115, 3508 TC Utrecht, The Netherlands; 2 Department of Palynology and Climate Dynamics, University of Goettingen, Untere Karspüle 2, 37073 Goettingen, Germany; 3 Lower Saxony Institute for Historical Coastal Research, Viktoriastr. 26/28, 26382 Wilhelmshaven, Germany; 4 Institute for Plant Sciences and Oeschger Centre for Climate Change Research, University of Bern, Alternbergrain 21, CH-3013 Bern, Switzerland; 5 IMBE-CNRS, Aix-Marseille Université, IRD, Avignon Université, Technopôle Arbois-Méditerranée, Bât. Villemin – BP 80, F-13545 Aix-en-Provence cedex 04, France; 6 Geography Department, University of Utah, 260S. Central Campus, Salt Lake City, UT USA

**Keywords:** Biogeography, Climate-change ecology, Palaeoclimate, Biodiversity

## Abstract

Climate warming is expected to cause a poleward spread of species, resulting in increased richness at mid to high latitudes and weakening the latitudinal diversity gradient. We used pollen data to test if such a change in the latitudinal diversity gradient occurred during the last major poleward shift of plant species in Europe following the end of the last glacial period. In contrast to expectations, the slope of the gradient strengthened during the Holocene. The increase in temperatures around 10 ka ago reduced diversity at mid to high latitude sites due to the gradual closure of forests. Deforestation and the introduction of agriculture during the last 5 ky had a greater impact on richness in central Europe than the earlier climate warming. These results do not support the current view that global warming alone will lead to a loss in biodiversity, and demonstrate that non-climatic human impacts on the latitudinal diversity gradient is of a greater magnitude than climate change.

## Abstract (web summary)

Climate-induced poleward shifts in plant distributions could flatten latitudinal diversity gradients. However, here the authors show that the spread of forests after the last ice age reduced diversity in central and northern Europe, and that human land-use over the past 5000 years strengthened the latitudinal gradient in plant diversity.

## Introduction

The strength of the correlation between modern spatial patterns in floristic diversity and climate variables^1^ suggest that a change in climate, such as current global warming, should result in a shift in the geographical distribution of biodiversity^2^. Global biodiversity patterns are shaped by speciation, extinction and migration^3^. While global warming may have long-term effects on the rate of speciation^4^, the immediate effects will be due to extinction and migration^5^. Attempts to understand and quantify these effects have been limited to predictive numerical models of species distributions given the brevity of the observational record of floristic diversity at these scales. The last time our planet underwent similar rates of warming was at the end of the last ice age^6^, and this period, for which a large amount of paleoecological data has been analysed and assembled, provides a relevant parallel for studying the reaction of the biota to current global warming.

Palaeoecological and genetic data from Europe and North America show that many trees spread into higher latitudes after the last ice age^7–11^. In North America, the southern distribution limits also shifted north for many trees with postglacial warming^11^, but not in Europe^10^. These poleward shifts in distribution limits have been observed for some biota with recent climate warming^12^ and are interpreted as plants tracking their climatic niche. This information has been used to drive correlative species distribution models (SDMs), which suggest that many taxa were restricted to southern Europe with the colder climate during the last glacial maximum (LGM), around 20 ka ago^13^. This concentration of species at lower latitudes resulted in a strong latitudinal gradient in species richness. As temperatures rose at the end of the last glacial period, many taxa shifted poleward, enriching the northern species pool. Coupled with a reduction in the southern pool, this would have weakened the latitudinal gradient in richness. This assumption of species distributions tracking the latitudinal shifts in climate underlie many concepts and studies of the reaction of biota to climate warming^14^, although it is rarely explicitly stated but see^15^. In addition to the zonal changes, higher taxonomic and compositional turnover are expected in the coastal western part of central Europe where climate change was more pronounced than in the continental interior^16^.

Evaluating these hypotheses requires detailed knowledge on species distributions between the LGM and present. Estimating species distributions using models with simulated LGM climate is insightful, but has many sources of uncertainty, including the climate simulations^17^, no-analogue climate configurations^18^, and the fact that SDMs can only predict climate suitability and not actual presence. Fossil pollen data represent an alternative, but underused, source of information on past changes in biodiversity. They contain information on plant richness^19^, and there are a large number of records from lakes and bogs for the period following the end of the LGM until the present, providing an almost continuous record of past vegetation change. Here, we use the European Pollen Database (EPD) to analyse postglacial changes in pollen-type richness and diversity across Europe. In particular, we examine how the latitudinal diversity gradient of vascular plants changed with postglacial warming, and how this matches the expectations detailed above.

Pollen identification yields different taxonomic levels: rarely species, but often genera and sometimes only family level. The relationship between pollen and plant taxonomy is a form of higher-taxon surrogacy^20^ and comparative studies have shown that spatial patterns in plant diversity are captured by pollen^21–24^. To evaluate the effect of higher-taxon surrogacy in this study we explore these patterns at two hierarchical levels based on pollen morphology following harmonisation of the full dataset. As the number of pollen types encountered per sample increases with sample size, we use rarefaction analysis to remove this bias and compare site-based data at a common, lower sample size of 500 identifications^25^. Samples were pooled for large regions in order to amplify the signal from herbaceous plants that generally have lower abundances and/or pollen production. Regional pollen type richness was compared based on a sample size of 50,000 identifications. While it is impossible to differentiate between locally produced and long distance transported pollen^26^, the number of prolific pollen producers is small. As these are present at nearly all sites they have little overall influence on the regional patterns analysed here. Finally, we compare trends in pollen type richness with indices of the overall change in regional pollen sample composition based on the first axis of a detrended correspondence analysis (DCA) as well as its constrained form (DCCA) with time as a constraint. These provide an estimate of community turnover by showing the timing of changes in vegetation composition^27^.

We find that the poleward spread of plants following the last ice age did not result in a weakened latitudinal gradient in pollen type richness. Increased temperatures at the onset of the Holocene did not increase the regional pool of pollen types in central and northern Europe. At individual sites pollen type richness declined as open vegetation types were replaced by forests strengthening the latitudinal gradient until 7 ka. We observe an increase in regional and site-based richness in central and northern Europe in the Late Holocene, as the manmade opening of the canopy during the last 5 kyr provided space for a rich herbaceous flora and the spread of agriculture introduced new species. Our results suggest that rapid climate warming, as documented for the onset of the Holocene in Europe, does not lead to an increase in richness at higher latitudes. The legacy of several thousand years of agriculture has a greater impact on continental scale diversity patterns than climate-induced shifts in plant distributions.

## Results

### A stable regional gradient with postglacial warming

Taxonomic harmonisation of the EPD yielded 860 pollen types (H_0_) and 310 types at the highest taxonomic level (H_2_). Patterns and trends obtained for all analyses were similar regardless of the taxonomic level, indicating that they are robust and little affected by pollen taxonomical biases. We, therefore, focus on results for the most detailed set (H_0_), unless stated otherwise. A comparison between the results for H_0_ and H_2_ is provided in supplementary Figs. 1, 3A, 5, 7 and 8A, and Supplementary Table 1.

**Fig. 1:**
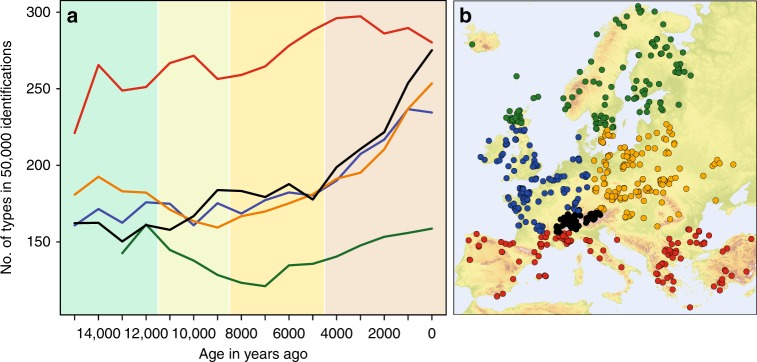
Regional pollen type richness through time based on 749 pollen diagrams from Europe and Turkey. **a** Pollen type richness in regionally pooled samples with up to 10^7^ identifications per sample. Comparisons are made at a sample size of 50,000 identifications using rarefaction based on all accepted types. Standard deviations are not shown due to their small values between 3 and 9. Line colours indicate regions: green = Boreal, blue = Temperate Oceanic, orange = Temperate Continental, red = Meridional/Submeridional, black = Alps. Time-periods (rounded to the nearest 0.5 ka) indicated by background colours: Lateglacial—11.5 ka—Early Holocene—8.5 ka—Middle Holocene—4.5 ka—Late Holocene. **b** Locations of pollen diagrams used in the analysis. The colouring of the locations corresponds to the line colours of the regions in (**a**). Digital elevation data for Fig. 1b was obtained from the European Environment Agency (EEA).

**Fig. 2:**
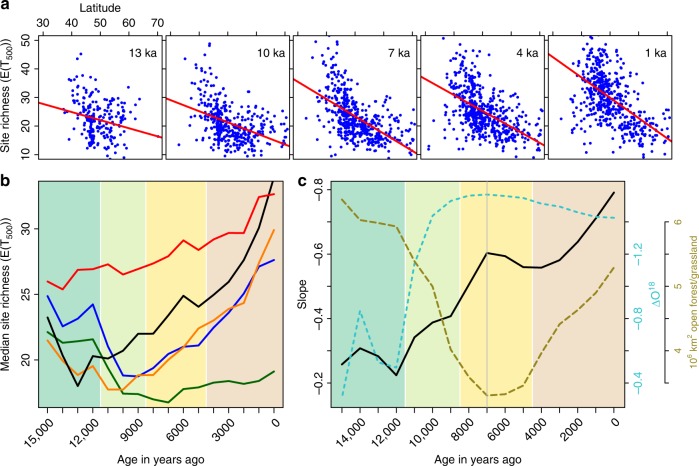
Site-based changes in pollen type richness at a standardized sample size of 500 identifications. **a** Scatter plots of pollen type richness versus latitude for selected time slices. **b** Median richness over all sites per region, colour coding as in Fig. 1. **c** Slope of the regression between pollen type richness and latitude of the site = black line; NGRIP Oxygen isotope record^28^ resampled to the same resolution = light-blue broken line; area of partially open forests and grasslands in Europe taken from ref. ^10^ as a measure of landscape openness = brown broken line.

A clear latitudinal gradient in richness is apparent in the regionally summarised samples, with a consistently higher level of pollen type richness in the Meridional/Submeridional and consistently lower level in the Boreal region (Fig. 1, Supplementary Figs. 1 and 2). However, the changes in this gradient do not match the expectations described above. Regional pollen type richness does not increase during Early Holocene warming around 10 ka ago (Fig. 2) in the Temperate and Boreal regions. The Meridional/Submeridional region shows a small increase in pollen taxa, while a decline due to the northward shift of tailing edges of distributions was expected. A delayed increase can be observed for the Alps, which may indicate the recolonization of this previously glaciated mountain range. Pollen type richness declines in the Boreal and Temperate Continental regions with minima at respectively 7 ka and 9 ka ago. In the last 3 ky, pollen type richness increases in the two Temperate regions and the Alps. If we consider the difference in the number of pollen types in the Meridional/Submeridional versus the Temperate and Boreal regions to represent the latitudinal gradient, this increases at 11 ka, reaches its maximum strength at 4 ka and declines over the last 3 ky (Supplementary Fig. 3).

### The site-based gradient increased with postglacial warming

This latitudinal gradient in pollen type richness is also present at the site level (Fig. 2, Supplementary Figs. 4 and 5) with an increase over the Holocene (the last 11.5 ky BP). During the Lateglacial period (15–11.5 ka BP) there was little difference in pollen type richness between the Mediterranean and central Europe, resulting in a flattened gradient. The slope of the gradient increases during the warmer Allerød period (14.5–13 ka BP) and then decreases during the cooler Younger Dryas period (13–11.5 ka BP). With the beginning of the Holocene, the slope of the gradient increases over the next 3 ky follows the increase in hemispheric temperatures recorded by the Greenland ice cores^28^. A first maximum is reached at 7 ka, at which time the regional diversity in the Boreal region is at a minimum, which equally corresponds to the period of densest forest cover in Europe (Fig. 2c; 10). The slope of the gradient increases again over the past 3 ky, driven mainly by the increased richness in Temperate sites and little change at Boreal sites (Fig. 2b). In central Europe, this corresponds to an accelerated opening of the forest cover around 3 ka^29^, around 4 ka later than the first spread of cereal cultivation^30^, which occurred at the time of densest forest cover. While the first increase in the gradient coincides with forest closing and competitive replacement of plants, the second increase is associated to the opening of the forest in Temperate regions. Over the last 2000 years, site-based richness increases at a faster rate than the Temperate and Meridional/Submeridional regional trends. This indicates a decline in spatial beta diversity, with sites becoming more floristically similar (Supplementary Fig. 6).

## Discussion

**Fig. 3:**
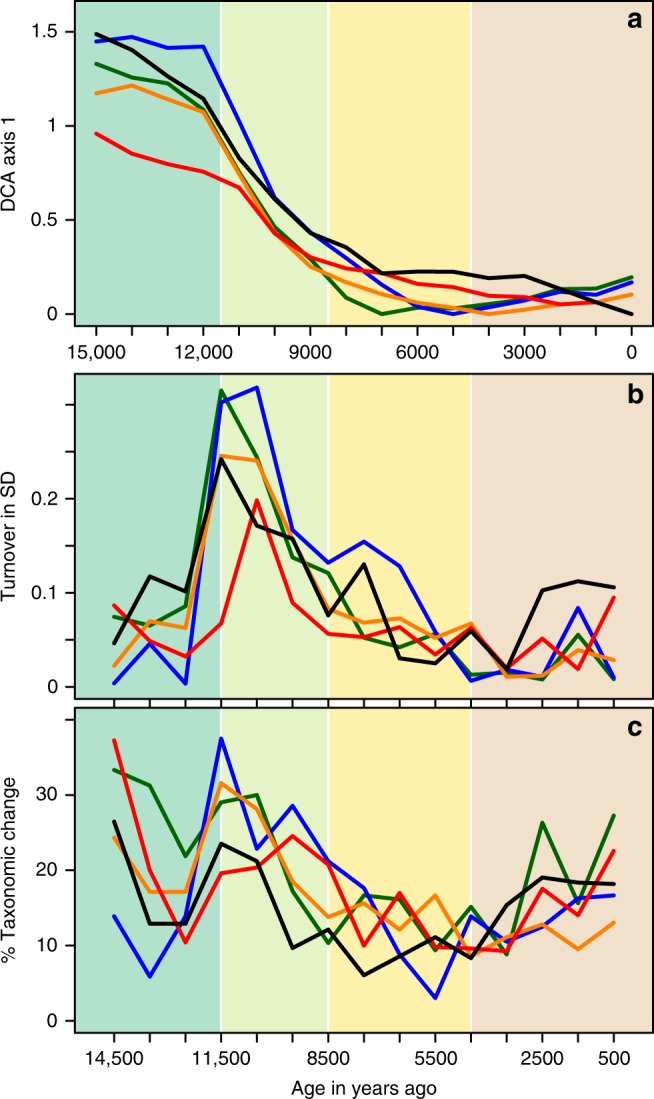
Compositional and taxonomic turnover of regionally combined samples assessed through detrended correspondence analysis (DCA), its constrained version (DCCA) and the number of different taxa in adjacent time-slices. Colour coding as in Fig. 1. **a** Scores of the first DCA axis, summarising the overall change in composition including abundance changes. Note that the gradient is shortest for the Meridional/Submeridional region and that samples from the Boreal and Temperate regions dating between 7 and 4 ka mark the end of the gradient, which is more pronounced when rare taxa are ignored (Supplementary Fig. 7, Supplementary Table 1). **b** Between-sample differences in the scores of the first, time constrained DCCA axis indicating that the highest compositional change between any two samples occurred at the onset of the Holocene in the Boreal and Temperate Oceanic regions. **c** The percentage of taxonomic change between consecutive samples of taxa with an abundance >0.1%, with taxa harmonised at a higher taxonomical combination H_2_ (see Supplementary Fig. 8 for H_0_).

Although little change was shown in the regional pollen type richness prior to 5 ka, the vegetation cover in Europe underwent a major reorganization during the Early Holocene (11.5–8 ka). Ordinations of the regionally summarised samples (Fig. 3) show a rapid shift in vegetation composition within the first 2 ky following the onset of climate warming 11.5 ka ago, driven by the rapid spread of trees (reaching more than 1000 m a^−1^)^31^. At the regional scale, this Early Holocene change in vegetation cover is primarily driven by changes in abundances and to a lesser extent by changes in floristic composition; changes in taxonomic composition are less pronounced and only discernible when ignoring scarce occurrences (Fig. 3c). Despite the model of poleward movement with warmer temperatures, many elements of the arctic-alpine flora that were abundant in central Europe during the LGM were not lost at once but persisted in some southern locations well into the Holocene. These are to be found on bogs, e.g. *Betula nana*^32^, above the tree-line and in other naturally open habitats, e.g. coastal areas^33^. The inclusion of sites representing these areas explains why the regional pollen type richness (Fig. 1) does not decline in the Alps and the Temperate Oceanic region with the spread of trees at the beginning of the Holocene.

During the last glaciation, European vegetation was not only limited by lower temperatures, but also by reduced water availability. This resulted in the dominance of herbaceous vegetation types throughout the continent, with trees restricted to moist mountain slopes and river valleys in central and southern Europe^34^. The Glacial and Lateglacial flora of central and north-western Europe likely contained many elements that are ascribed today to a ruderal flora^35^. With the spread of forest during the Early Holocene, these light demanding plants lost most of their habitat, but a large proportion of this flora reappeared following deforestation in the Late Holocene^36^. Establishing whether the same species occur in central Europe today as in the LGM is made difficult by limits on the taxonomic resolution of these plants; identification is generally only possible to the genus level. However, several examples support this scenario, e.g. the pollen type associated with *Plantago lanceolata* has been recorded in north and central Europe during the Lateglacial. Pollen types including *Papaver argemone*, *Sanguisorba minor* or *Centaurea cyanus* were found in Lateglacial sediments in and around Berlin. These subsequently disappear from Early to Middle Holocene samples and reappear with the later deforestation of the area^37^. More examples are available for the British flora^35^ including again, some that are not present during the Early or Middle Holocene, but are found before and after. The opening of `the forest for agriculture in the second half of the Holocene also provided new habitat for plants that were deliberately or accidentally introduced with farming (archaeophytes). Few archaeophytes can be unambiguously identified by pollen so it is difficult to estimate their contribution to this increase in richness. These have, however, made a significant contribution to the central and north-west European floras^38^. This continental scale anthropogenic landscaping resulted in increased regional and site-based pollen type richness during the Late Holocene, seen most clearly in the temperate regions and the Alps.

It may be argued that the strong Late Holocene increase, as well as the lack of an early Holocene increase in pollen type richness, are an artefact connected to the expansion and reduction of high pollen-producing taxa drowning the signal of herbaceous pollen. However, the forest did not close instantaneously at the beginning of the Holocene; open areas persisted for a few thousand years^29^. If herbaceous plants spread at the same rate as trees, a peak in pollen type richness should have been observed before the forest cover closed.

**Fig. 4:**
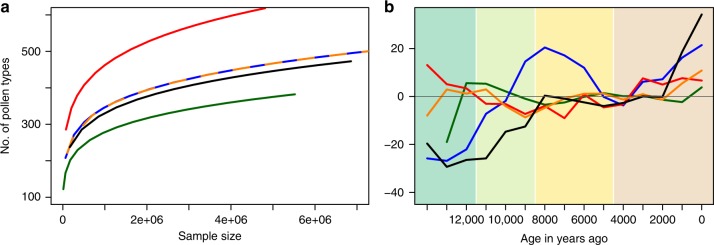
Taxon accumulation for the regionally summarised samples on all accepted types H_0_. **a** Power functions describing the overall trend of a common sampling effect (encounter of new taxa with increased sample size) for regions with different richness. **b** Residuals of the regional taxon accumulation curves (actual values minus the trend in A) plotted against time. The oldest samples show lower than expected numbers of taxa due to a low number of sites and were omitted for clarity. Colour coding as in Fig. 1.

All regional taxon accumulation curves show the same general increase in the number of unique pollen types found as the sample size (sampling effect) increases following a power law (Fig. 4). Deviations from a modelled function show that the number of taxa in the Alps and Temperate Oceanic region increased rapidly before 8 ka BP, which would be expected with the postglacial immigration of new species. In contrast, number of taxa found in the Temperate Continental region are close to the expected value. Changes in limiting climate conditions may have been less severe in this region, as reflected in the relatively small compositional changes (Fig. 3). This would, in turn, mean that the Lateglacial and potentially LGM flora in central Europe contained more taxa than current northern European regions, despite having similar mean annual temperature. The interannual temperature variability was several times larger during the LGM compared to the Holocene^39^ and it is conceivable that annual taxa would have been able to persist if suitable weather conditions occurred sporadically and a good seed bank existed. Similarly, perennial taxa may have survived in sheltered places with regeneration limited to periods of uncommonly high temperatures. Phylogeographic studies have shown the persistence of herbaceous and shrubby species in areas north of the Alps during the LGM^40,41^. A recent analysis of the current distribution of the European flora suggests that many species in Europe did not spread with Holocene warming, despite climatically suitable regions becoming available^13^. Thus, while many of the trees have been restricted to southern locations during the LGM, a large proportion of the European flora may have persisted further north during glacial interglacial cycles, changing abundance as climatic conditions altered rather than distribution.

Although pollen records represent a biased and incomplete account of the past vascular flora, they represent one of our best data sources to evaluate continental scale changes in plant diversity and richness gradients outside of the observational period, with postglacial climate warming representing the most recent analogue for a planetary-scale critical transition^42^. Our analysis suggests that while this warming resulted in a significant reorganisation of vegetation composition and structure, the impact on regional richness patterns was comparably small. The assumption that most plants shifted their ranges with postglacial warming is well founded for many tree species but this model may not hold for a large proportion of the European flora. Rather than shifting distribution limits, many plants in central and southern Europe may have reacted by changing abundance or through local extirpation with the expansion of temperate forests. In contrast, the opening of the landscape during the spread of agriculture had a much greater impact on the regional richness patterns, increasing richness in the central part of Europe and reducing the differences between regional pollen type richness. Overall, these results suggest that rapid climate change does not lead to correspondingly rapid shifts in continental scale diversity patterns. In comparison, the legacy of several thousand years of agriculture and the introduction of new taxa have had a much greater impact on diversity patterns. Different life strategies, landscape heterogeneity as well as interannual climate variability need to be considered in order to improve projections of future diversity changes.

## Methods

### Taxonomic harmonisation

When comparing the number of taxa across studies from different investigators it is essential to harmonise names identifying the same morphological type (synonyms). Different European traditions in pollen taxonomy give rise to the problem that similar types may be lumped together or split up into many different types. Both synonyms and differences in the taxonomic detail, were harmonised for this study. We extracted the full taxon list stored in the EPD, systematically checked each of the over 4000 names used for pollen and spore types, and assigned an EPD-accepted variable name. This EPD-accepted taxon name represents the currently accepted most detailed level of pollen identification according to the literature and expert knowledge. Taxon names suggesting higher taxonomic detail were assigned to this level, even where this meant losing presumed taxonomic resolution, resulting in about 1200 EPD-accepted taxon names. This taxonomical level (H_0_) generally links the pollen type to groups of closely related plant species, which may represent single species in species poor regions. Based on the EPD-accepted taxonomy (H_0_) we constructed two higher hierarchical levels (H_1_ and H_2_) based on pollen morphological features reflecting different levels of taxonomic precision. In the first higher level (H_1_) we combined morphologically similar types that may be separated only where a good reference collection is available, which resulted in about 800 different taxa generally referring to the genus or closely related genera in plant taxonomy. The second hierarchy level (H_2_) combines types with distinctive, readily identifiable features often corresponding to related groups of plant genera or the plant family level, and this reduced the number of types to about 400. As the EPD includes sites outside the European continent, the number of taxa included in the analyses was reduced to 860 for H_0_ and 310 for H_2_ after matching the variables of the selected sites with these lists. The synonym and hierarchy table is available as a csv file from http://www.europeanpollendatabase.net/data/downloads/.

### Site selection

We used the subset of sites in the EPD with standardized age models^43^ and constrained the area of investigation to sites between 25°W and 35°E longitude and north of 35°N latitude. This includes sites east of the Mediterranean, which is the source for cultivated cereals and potentially for weeds introduced to central Europe with the spread of agriculture. Within these boundaries, we divided the sites into five regions with the aim to differentiate between broad regions with different climate and vegetation history, while creating subsets of similar size. Sites at latitudes higher than 57°N were assigned to the Boreal and sites south of 45°N to the Meridional/Submeridional region. The Temperate region was divided into oceanic and continental at 11°W. The Alps were singled out as a separate region due to their climatic heterogeneity but also due to the high number of sites available from this area, including sites located between 45°N and 47°N and 5°W to 15°W. The resulting set of sites include some diagrams with limited identification of herbaceous pollen types, which we excluded from further analysis by considering only pollen sequences with at least 32 identified pollen types. This threshold was found by analysing the frequency distribution of the number of identified types per sequence, which had a minimum near 32 identifications. In addition, 32 identifications corresponds to the average number of identified pollen types in samples from species poor environments like the boreal forest. This constraint reduced the total number of pollen sequences included in the analysis to 749.

### Data analysis

Pollen types identified in these sequences were matched with the three pollen taxonomies defined above (H_0_, H_1_ and H_2_) for all terrestrial pollen and spores of vascular plants, summing counts where the assigned variable matched more than one original variable name. As initial analyses, using H_1_ yielded results that were intermediate between H_0_ and H_2_, this dataset was not included in further analysis. For the two other datasets, samples in each sequence were binned into samples representing 1000 years centred on full 1000-year intervals for the last 15,000 years. If the resulting samples contained less than 500 pollen counts, these samples were excluded from site-based analysis, but included in the regional pool. Regional samples were obtained by summing pollen identifications from samples for a region and time. We also compiled the data into tables containing all samples per region and time slice. This data handling was carried out in R using the reshape2 library^44^.

As the number of types encountered increases with the sampling effort, comparisons across different samples and sites require a standardized sample size. To correct for this, we use rarefaction analysis with the function rarefy in the vegan package^45^. In the analysis of taxonomic turnover, we used sample based percentage thresholds to decide on the inclusion of a rarely occurring taxon in a particular sample. For these cases, pollen percentages were computed based on all terrestrial pollen and spores. A sum of 50,000 grains was used for rarefaction of the regional samples. We compared the regional samples for all terrestrial pollen and spore types as well as for trees and shrubs only (Supplementary Figs. 1 and 2) and computed the difference in the number of taxa between regions (Supplementary Fig. 3). Between-sample differences in taxonomic composition (Fig. 3c, S7) were evaluated as β_cc_ = (b + c)/(a + b + c), with a = taxa occurring in both samples and b, c = taxa occurring only in one of the two adjacent samples. Results were multiplied by 100 to be expressed as percentage change. Time constrained and unconstrained detrended correspondence analysis (DCCA, DCA) were carried out (Fig. 3) for the regional samples treating each region independently, applying square root transformation and detrending by segments using Canoco 5^46^. Taxon accumulation curves are based on the cumulated increase in taxa versus pollen sums over consecutive samples, generally resulting in linear relationships in log–log space^37^. Here, we log10 transformed both accumulated pollen counts and accumulated taxon number, then fit linear trends to the resulting transformed data. A common slope of 0.185 was empirically derived (Supplementary Fig. 9). The residual for each time step was used to examine the time dependent deviation from the expected number of taxa in each region.

For site-based analyses, we used a common pollen sum of 500 grains for rarefaction. The rarefied taxon numbers were then used to estimate the latitudinal gradient of diversity for each time step, by regressing these taxon numbers against the latitude of the site. The coefficient of determination (R2) and the slope of the regression curve were then used to explore the relative strength of the gradient over time. For visual comparison we plotted the changing slope through time together with the NGRIP Oxygen isotope record^28^ as an indicator of hemispherical temperature change and area of partially open forests and grasslands in Europe to describe the general changes in landscape openness across Europe. This curve represents the area in Europe for which a pollen deposition of more than 4% Poaceae pollen was interpolated^10^. We also computed the latitudinal gradient based on the absolute number of pollen types per samples irrespective of sample size (Supplementary Fig. 5). Beta diversity was also estimated from the rarefaction analysis as the median of the rarefied regional sample divided by rarefaction to 500 of all site samples contributing to the regional pool. An estimate of regional beta diversity (Supplementary Fig. 6) was calculated as the median ratio between the regional richness (rarefaction to a sample size of 50,000 Fig. 1) and the site-based richness (rarefaction to a sample size of 50,000 Fig. 2).

### Reporting summary

Further information on research design is available in the Nature Research Reporting Summary linked to this article.

## Supplementary information

Supplementary Information

Reporting Summary

## Data Availability

All analyses were carried out using the 2015 Access version of the EPD, which together with the synonyms and hierarchy table is freely available from http://www.europeanpollendatabase.net. Age models for the sites are available from Pangaea: 10.1594/PANGAEA.804597.
