# Supplementary material for: Postglacial change of the floristic diversity gradient in Europe
Source: Nat Commun. 2019 Nov 28;10:5422. doi: 10.1038/s41467-019-13233-y (PMC6882886; doi:10.1038/s41467-019-13233-y)
Supplement: Supplementary file 1 — Supplementary Information [file 41467_2019_13233_MOESM1_ESM.pdf]

Supplementary Information

Postglacial change of the floristic diversity gradient in Europe

Giesecke et al.

Supplementary Notes

As several choices were made in defining the data set and taxonomic level used in the analysis, the following account provides additional information on the robustness of the trends and patterns observed.

Supplementary figures 1, 2 illustrate the trends in regional or pollen-type richness based on different taxonomic resolutions (H_0 and H_2). Despite the difference in resolution, there is little difference in the reconstructed trends. The number of available samples and the size of pollen counts per sample increase with time in all regions (panel c in Suppl. Fig. 1, 2), which determines a large portion of the variation in pollen type richness, emphasizing the need for standardization through rarefaction analyses or percentage thresholds. Nevertheless, some of the features in regional pollen type richness are so robust that they are visible even without standardization. One of these features is the rank order of regional pollen type richness, which is always highest in the Meridional/Submeridional region even though pollen counts for this region are among the lowest available. In addition, the decline in the number of pollen types in the Boreal region after the beginning of the Holocene is also visible in the absolute number of types. A comparison of the trends based only on pollen types from trees and shrubs with the total number of pollen and spore types shows that the overall signal is mainly coming from herbaceous pollen types. Many trees produce abundant pollen, which is generally dispersed over long distances so that pollen types from the abundant European trees occur in most samples regardless of region or time. Nevertheless, the rank order of regional pollen type richness for tree and shrub pollen is the same as for the full set of pollen types and the curves for the Temperate regions and Alps show a rise with the beginning of the Holocene (panel d in Suppl. Fig. 1, 2). The tree and shrub curve for the Boreal region shows an interesting maximum at 6 – 5 thousand years ago (ka).

The differences in the number of pollen types in the Meridional/Submeridional versus the Temperate and Boreal regional samples remain relatively constant over time (Suppl. Fig. 3). A small rise in the gradient occurs at the beginning of the Holocene with a decline for the last few thousand years. Much of the variability in the regionally combined samples is driven by the occurrence and frequency of rare pollen types, which occur sporadically throughout the sequences. About 30% and 20% of the taxa change between all adjacent regional samples for H_0 and H_2 respectively, without large differences between the regions and without any temporal trend. A temporal trend in taxonomic turnover emerges only when rare taxa are excluded. At sample-based thresholds between 1% and 0.001%, we observe a peak in regional pollen type turnover after the onset of the Holocene (Suppl. Fig. 7). The timing of this peak varies depending on which threshold and dataset are used, but the following general trend is persistent: higher compositional change in the Lateglacial, an Early Holocene peak, a Middle Holocene trough, and a smaller Late Holocene rise.

The Detrended Correspondence Analysis (DCA) provides another way to assess turnover between samples in the regional datasets including abundance changes. The short ordination gradients demonstrate that taxonomic turnover was small at the regional scale. The gradient is longer if rare taxa are excluded, regardless of the taxonomic harmonisation used (Suppl. Fig. 6, Suppl. Table 1). This supports the idea that these less abundant taxa occur infrequently and sporadically throughout the time covered, as the gradient would lengthen if these were restricted to particular time-periods. The gradient in sample scores is always shortest for the

Meridional/Submeridional samples, which reflects the smaller amount of change in the species composition of this region. The changes in sample scores along the first DCA axis clearly show that shifts in overall regional vegetation composition started with the onset of the Holocene and lasted until 7 or 8 ka, which is in agreement with the timing of the first peak in the slope of the diversity gradient.

Taxon accumulation curves (Suppl. Fig. 8, 9) describe the relationship between sample size and number of pollen types, allowing a comparison of the absolute number of types in the regional samples. Plotted in log–log space, the five regions follow linear trends with similar slopes and different y-intercepts (Suppl. Fig. 8), indicating that while there are baseline differences in the number of taxa, the sampling effect is similar across regions. We fitted regression models with the same slope and different intercept and plotted the model residuals to identify periods where the observed number of taxa deviates from the expected value. Samples from the Boreal, Temperate Continental and Meridional/Submeridional regions follow this modelled trend, while the Alps and the Temperate Oceanic region deviate from this trend until 8 ka. Both regions start from a lower taxon number that rises faster than in the other regions until 8 ka (Suppl. Fig. 9). This deviation in the latter two regions would be consistent with the addition of new species with postglacial immigration, which was expected for all save Meridional/Submeridional region. However, the Alps were nearly completely glaciated leaving little room for the survival of plants and with Holocene warming many plants with nearby LGM distributions spread into the mountains. The Temperate Oceanic region changed to an area of mild winters supporting a flora that could not previously have survived there. In comparison, the changes in limiting climate conditions may have been of a smaller magnitude in the Temperate Continental region resulting a smaller compositional change (Suppl. Fig. 6). However, it is surprising that the samples from the Boreal region follow the general model that well; an effect of the postglacial spread of plants would be expected.

Robust trends are also seen in the latitudinal gradient of richness based on the site-level data. These trends are independent of taxonomic harmonisation and can be seen in the absolute number of different pollen types regardless of the pollen sum (Suppl. Fig. 4). The strength of the relationship declines in the second half of the Holocene, which is mainly due to the increased pollen counts in temperate regions. Nevertheless, the changes in the slope of the relationship between the absolute number of pollen types and latitude follow that of the rarefied number of taxa. For the most recent samples, this gradient suggests that from south to north one pollen taxon disappears per additional degree latitude.

Comparing the trends in median sites richness (at a pollen sum of 500) with the regional trends in richness (at a sum of 5^4), also provides information on between-site differences (Suppl. Fig. 5), with the two Temperate regions showing interesting trends. Between-site differences are highest in the Lateglacial and Early Holocene for the Continental region and peak at 9 ka for the Oceanic region, after which the curves decline in both Temperate regions.

Supplementary Figures

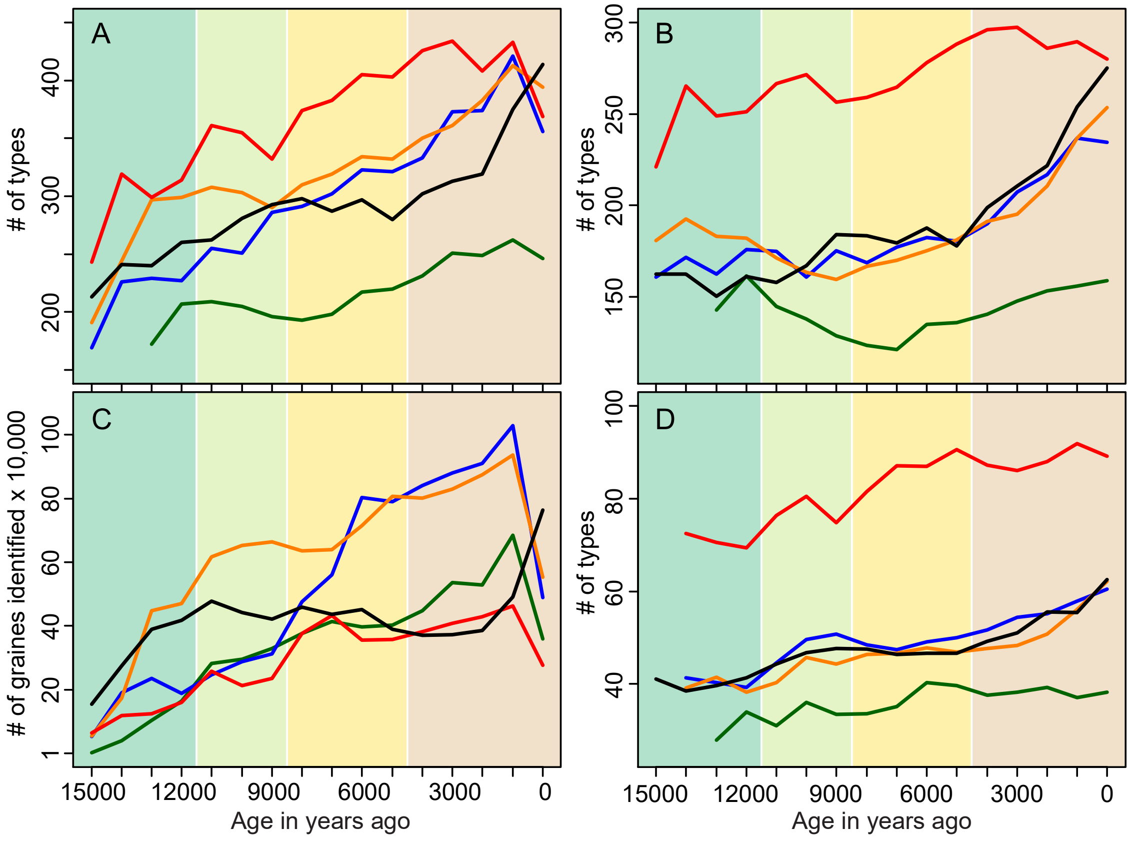

Supplementary Fig. 1. Pollen type richness in regionally pooled samples for H_0. A) Absolute numbers of pollen and spore types without adjustment for the sample size. B) Number of pollen and spore types in a sample of 50,000 identifications estimated by rarefaction. C) Number of pollen identifications per sample. D) Number of pollen types from trees and shrubs in a sample of 50,000 identifications estimated by rarefaction. Colour code for regions: green = Boreal, blue = Temperate Oceanic, orange = Temperate Continental, red = Meridional/Submeridional, black = Alps.

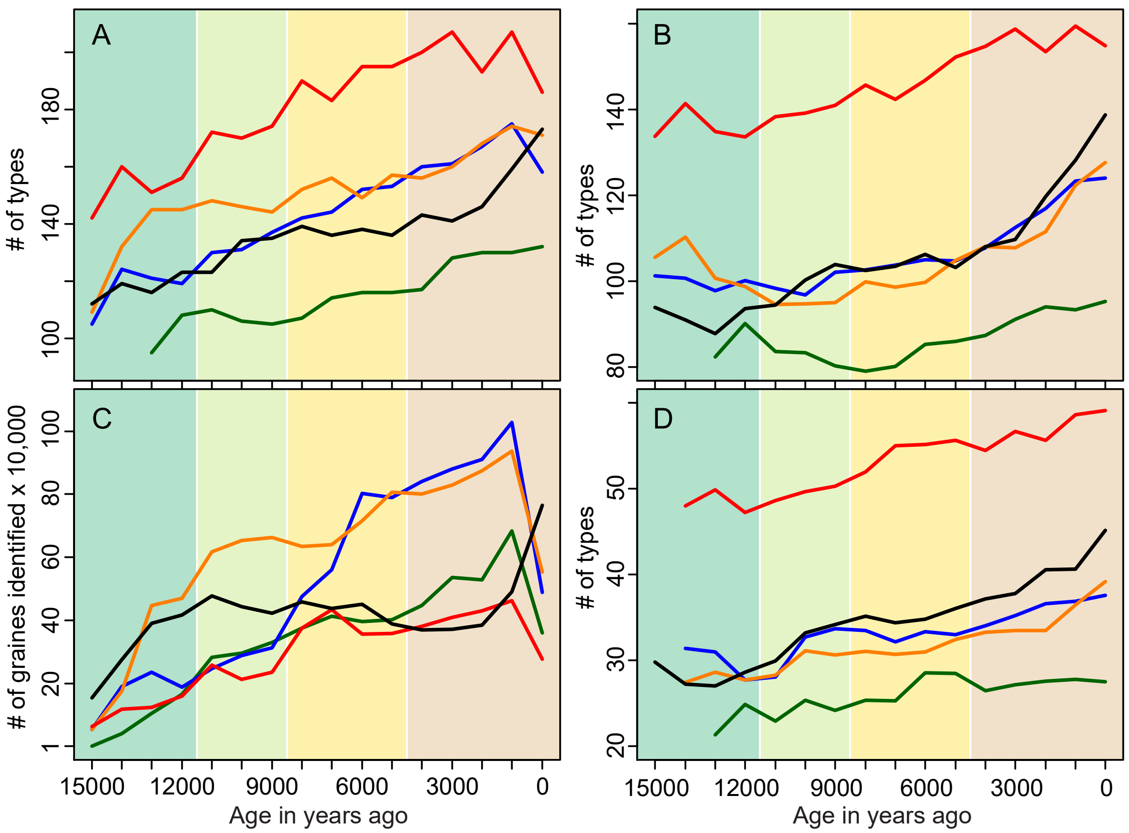

Supplementary Fig. 2. Pollen type richness in regionally pooled samples for H_2. A) Absolute numbers of pollen and spore types without adjustment for the sample size. B) Number of pollen and spore types in a sample of 50,000 identifications estimated by rarefaction. C) Number of pollen identifications per sample. D) Number of pollen types from trees and shrubs in a sample of 50,000 identifications estimated by rarefaction. Colour code for regions: green = Boreal, blue = Temperate Oceanic, orange = Temperate Continental, red = Meridional/Submeridional, black = Alps.

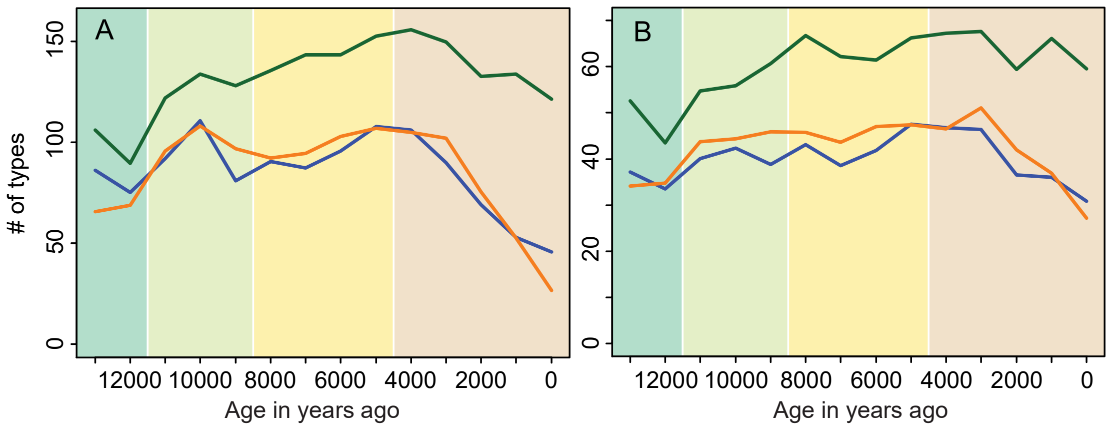

Supplementary Fig. 3. Difference in the number of pollen types in regionally pooled samples between the Meridional/Submeridional and Temperate as well as Boreal regions for A) H_0 and B) H_2, note the different y-axis scales. Colour code: green = Meridional/Submeridional - Boreal, blue = Meridional/Submeridional - Temperate Oceanic, orange = Meridional/Submeridional - Temperate Continental.

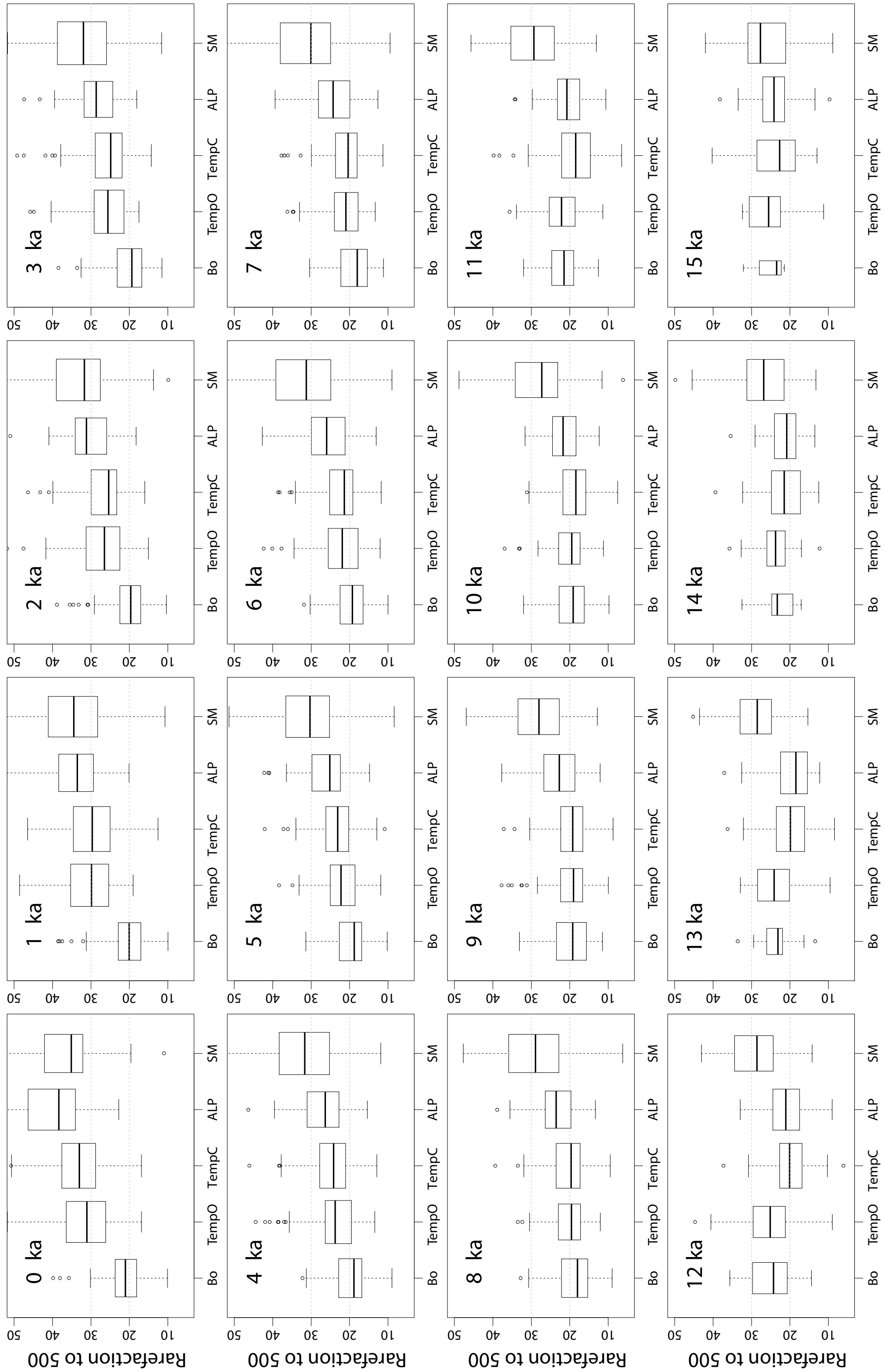

Supplementary Fig. 4. Box plots illustrating the regional scatter in site based palynological richness to the base count of 500 identifications for the time windows. The width of the box represents the number of sites. Bo = Boreal, TempO = Temperate Oceanic, TempC = Temperate Continental, ALP = Alps, SM =Meridional/Submeridional.

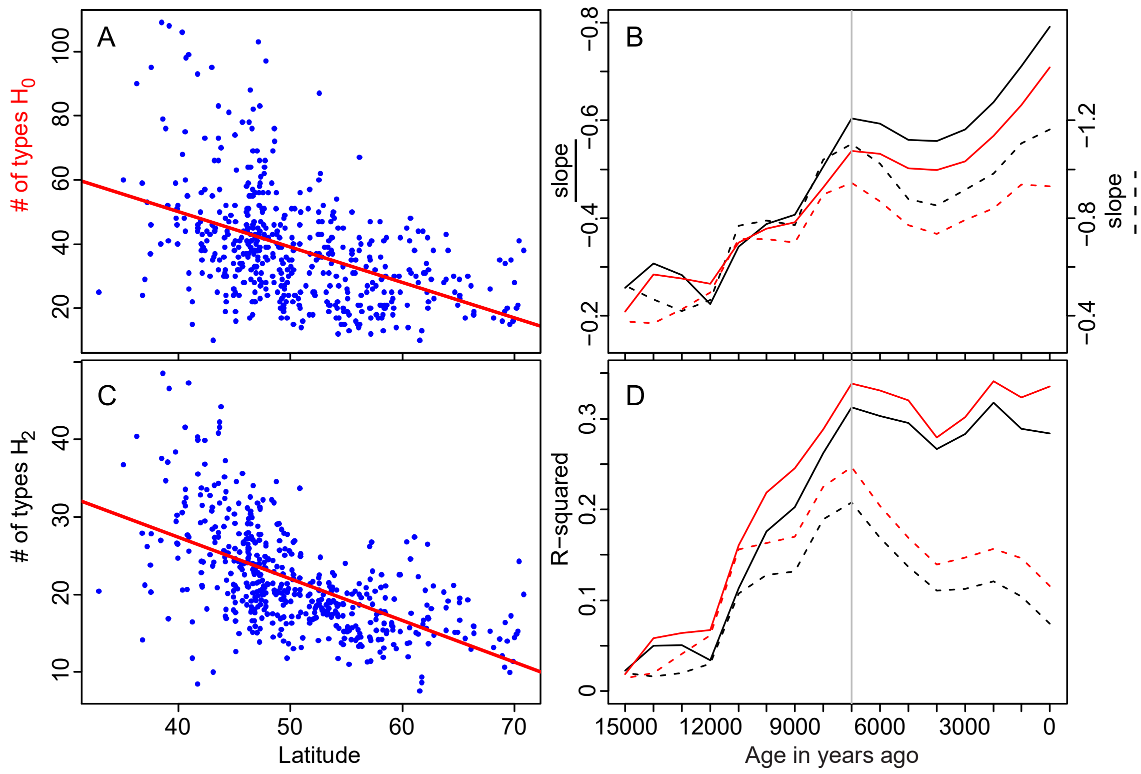

Supplementary Fig. 5. Latitudinal trends in site based pollen type richness. A), C) The gradient of palynological richness at a sample of 500 grains at 7000 years ago for the two hierarchical sets A = H_0, C = H_2. B), D) Changes in the gradient over the last 15000 years for H_0 = red and H_2 = black lines, based on a sample of 500 grains = continuous line and the complete sample = broken lines; C) changing slopes D) changing strengths of the relationship.

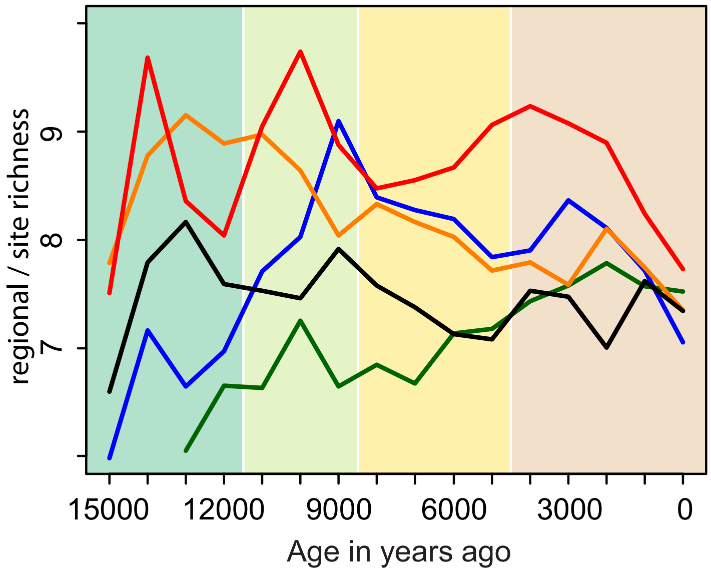

Supplementary Fig. 6. Between site differences in richness as the median ratio between the regional and site based richness per region and time based on H_0. Colour code for regions: green = Boreal, blue = Temperate Oceanic, orange = Temperate Continental, red = Meridional/Submeridional, black = Alps.

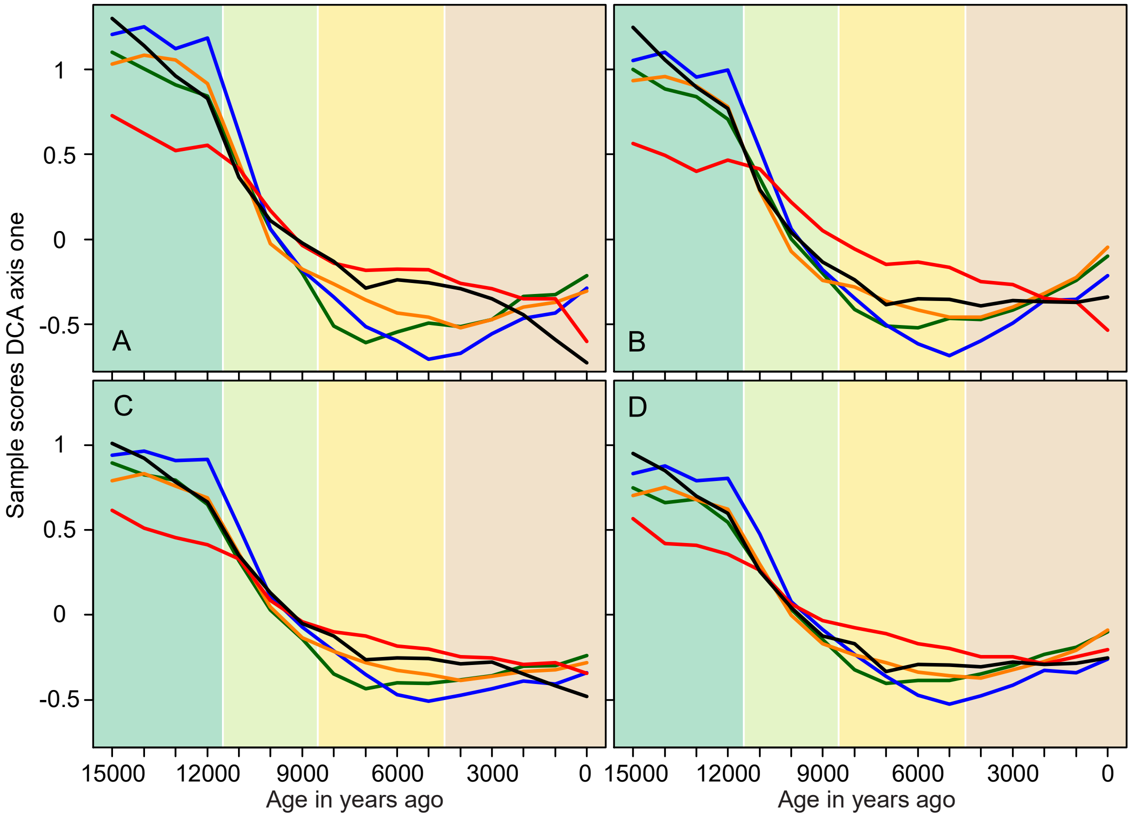

Supplementary Fig. 7. Turnover as estimated by the first axis scores of a DCA analysis A), B) with a threshold abundance of 0.1% for the inclusion of a taxon and C), D) without; A), C) using H_0 and B), D) using H_2 taxonomy. Colour code for regions: green = Boreal, blue = Temperate Oceanic, orange = Temperate Continental, red = Meridional/Submeridional, black = Alps.

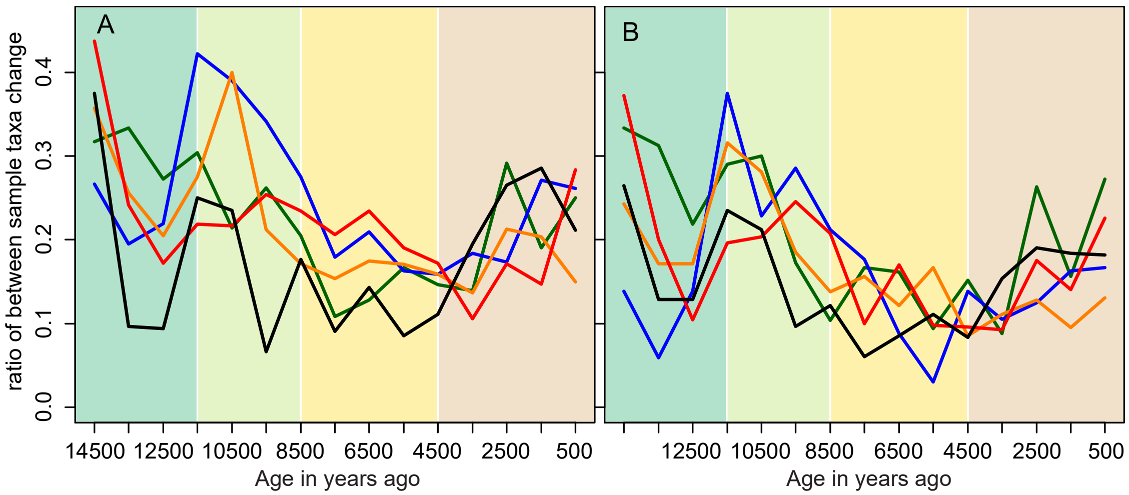

Supplementary Fig. 8. Change in the taxonomic composition as the ratio of taxa with an abundance > 0.1% changing between adjacent samples of the regional pool. A) H_0 and B) H_2. Colour code for regions: green = Boreal, blue = Temperate Oceanic, orange = Temperate Continental, red = Meridional/Submeridional, black = Alps.

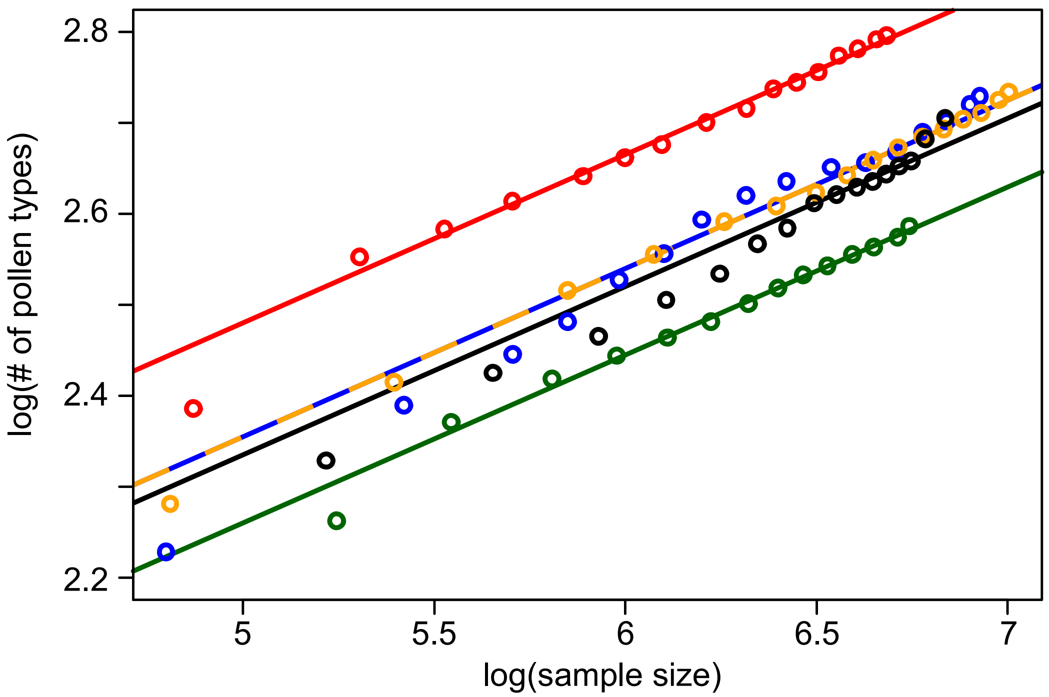

Supplementary Fig. 9. Taxon accumulation curve plotted with Log10 transformed values. Fitted linear relationships have a common slope, representing the sample effect and individual intercepts representing the differences in regional richness. Colour code for regions: green = Boreal, blue = Temperate Oceanic, orange = Temperate Continental, red = Meridional/Submeridional, black = Alps.

Supplementary Table 1. Gradient length in standard deviations along the first axis of the DCA of regional samples indicating the effect of the different taxonomical hierarchies (H_0 and H_2) and a sample based percentage threshold of 0.1% of the pollen sum for the inclusion of a taxon.

	**H_0**		**H_2**	
**Regions**	**all**	**0.1 %**	**all**	**0.1 %**
**Boreal**	1.36	1.65	1.10	1.42
**Temperate Oceanic**	1.64	2.01	1.47	1.75
**Temperate Continental**	1.34	1.67	1.13	1.41
**Meridional/Submeridional**	0.98	1.27	0.9778	1.11
**Alps**	1.46	1.84	1.26	1.6
